# A Narrative Identity Perspective on Mechanisms of Change in Imagery Rescripting

**DOI:** 10.3389/fpsyt.2021.636071

**Published:** 2021-12-16

**Authors:** Soljana Çili, Lusia Stopa

**Affiliations:** ^1^London College of Fashion, University of the Arts London, London, United Kingdom; ^2^School of Psychology, University of Southampton, Southampton, United Kingdom

**Keywords:** imagery rescripting, intrusive images, mechanisms of change, narrative identity, self-defining memories

## Introduction

Imagery rescripting (ImRs) is increasingly used in cognitive-behavioral therapy (CBT) to change beliefs and meanings about the self associated with negative and traumatic memories. It is quintessentially an imagery intervention that targets the self and autobiographical memory (AM); however, to date most of the research into its effectiveness has focused on symptom alleviation. The mechanisms of change remain unclear. In this article, we outline a narrative identity model of change in ImRs and note the value of the narrative identity literature in helping us understand memory-focused therapeutic interventions.

## ImRs as A Tool for Addressing Intrusive Mental Images in Clinical Disorders

ImRs describes a family of techniques in which patients recall and then imaginatively change a disturbing image/memory. The aims of this imagery manipulation can include any of the following: introducing a new perspective (e.g., seeing a childhood memory of sexual abuse from an adult perspective), contextualizing or adding information to the memory that was unavailable at the time (e.g., understanding that one survived despite believing that one would die during the trauma), seeing how the memory contributed to negative beliefs or experiences of the self, realizing how the memory contributes to distressing intrusive images, and activating a more understanding and compassionate attitude toward the self. The methods range from unstructured approaches exploring interventions such as bringing the adult self into the memory to structured approaches such as Arntz and Weertman's (1) three-stage protocol. The latter involves activating the memory in Stage 1. In Stage 2, the patient relives the memory again from the perspective of the adult self and is encouraged to intervene in any way s/he wishes. In Stage 3, the patient goes through the memory from the younger self perspective with the adult present so that the younger self can ask for any help that s/he needs but was not provided by the adult in Stage 2. Some applications of ImRs [e.g., (2)] use cognitive restructuring before embarking on ImRs that follows Arntz and Weertman's protocol.

Evidence suggests that ImRs is an effective intervention. A recent meta-analysis showed that it produced symptom alleviation and a significant reduction in the vividness of memory-related intrusive images and associated distress across a range of disorders (3). The minority of studies which looked at beliefs associated with the rescripted memories (the “*encapsulated beliefs”*) showed reductions following ImRs. Subsequently published research has examined the effects of ImRs in binge eating disorder (4), obsessive-compulsive disorder (5–7), major depressive disorder (8, 9), illness anxiety disorder (10), social anxiety disorder [SAD; (11, 12)], borderline personality disorder (13), psychosis (14), and voice hearers (15). Overall, this research supports previous findings on ImRs outcomes. Moritz et al. (8) also found improvements in self-esteem following a self-help ImRs intervention, although other researchers [e.g., (15)] failed to find this effect. Taken together, the existing studies suggest that memory and self-processes are central to ImRs. Current explanations of change focus on these processes.

## Existing Explanations of the Mechanisms of Change in ImRs

Explanations of change in ImRs exist at different levels. The most basic level focuses on associative learning processes and places the change in the meaning attached to the targeted memories/images at the heart of its therapeutic effects (1, 16–18). In this explanation, ImRs allows the reactivation of the distressing memories/images (the unconditioned stimuli), their emotional processing, and subsequent re-evaluation before they are reconsolidated. As a result of the re-evaluation, future activations of the rescripted unconditioned stimuli produce a new conditioned response (a diminished emotional response) which reflects the new meaning.

Higher-level explanations of change focus on the link between the change in meaning or encapsulated beliefs and the self. Mancini and Mancini (19) propose that ImRs contributes to meta-emotional changes. Seeing the experience from the perspective of the adult self allows the person to empathize with the younger self and initiates a change in the perception of the younger self's negative emotions. Specifically, earlier suffering may be perceived as “legitimate, adequate, and deserving of care” [(19), p. 3], resulting in acceptance of negative emotions instead of seeing them as problematic. This reappraisal may lead to reduced reactivity to these emotions, changes in self-representations, and symptom alleviation.

In a precursor to the model we describe below, Çili et al. (20) explained the effects of ImRs by drawing on the self-memory system (SMS) model (21, 22) and Brewin's (23) retrieval competition hypothesis. The SMS model proposes that the SMS initiates a search for a relevant self-defining memory when there is a shift in environmental demands that requires a response. The activation of this memory is accompanied by an affective response and the activation of a *working self* comprising goals and self-images. The retrieval competition hypothesis argues that cognitive-behavioral interventions like ImRs may contextualize aversive memories and make new or existing positive self-representations more likely to win the retrieval competition against negative self-representations (23, 24). Combining these two explanations, we proposed that ImRs facilitates the integration of aversive self-defining memories with individuals' other AMs by changing their meaning, and this may reduce the salience and accessibility of the memories and related working selves (20). As a result, the benign working selves gain a retrieval advantage in situations that would have previously favored negative working selves. According to our account, ImRs modifies the impact of the aversive memory on the working self and the reduced distress results from benign working self activation. In fact, Norton and Abbott (25) suggest that ImRs may reduce access to negative self-imagery and associated meanings and create new positive meanings or images. Our study showed that 1 week after rescripting a non-clinical sample rated their aversive memory as less negative, less distressing, and less important for their sense of self, reporting reduced post-retrieval state negative affect and higher state self-esteem and positive affect (20).

Despite encouraging evidence supporting the proposals above, the exact mechanisms operating in ImRs are yet to be uncovered. We argue that taking a broader picture of the memory-self relationship is critical to advancing the field and argue that the narrative identity literature provides a promising framework for understanding change in ImRs. Our expanded model is presented below.

## A Narrative Identity Model of Change in ImRs

We propose that understanding mechanisms of change in ImRs requires an understanding of AM functions and personality. First, AM serves at least 3 main functions: *directive* (guiding behavior, emotion, and cognition), *social* (developing and nurturing interpersonal relationships), and *self* (constructing and maintaining a sense of self) [e.g., (21, 22, 26–28)]. These functions can be adaptive or maladaptive (29) and this may vary across the lifespan (30). Second, McAdams [e.g., (31–33)] proposes that personality consists of three layers: *dispositional traits*; *characteristic adaptations* (e.g., goals, values, hopes, fears); and *the life story* or *narrative identity*, a constantly changing narrative about who one is, was, and may become which gives individuals a sense of unity, self-continuity, and direction in life. The life story allows individuals to integrate their emotions, cognitions, and behaviors so they can pursue their long-term goals (31, 32, 34). Germane to our model is evidence suggesting that, overall, life stories or AMs are associated with low levels of well-being or symptoms of psychological disorders when they are described with a low sense of agency (e.g., autonomy) and/or communion (interpersonal connection), are characterized by negative affect, and reflect contamination (shifts from a positive to a negative outcome) or negative connections between the memory and the self (e.g., “*This experience shows that I am a failure”*) [for reviews, see (35, 36); see also (37)].

We also propose that our understanding of ImRs may benefit from literature on the self and personality outcomes of therapy. This literature is relatively limited. A systematic review on self changes in CBT for SAD, for example, concluded that CBT produced significant reductions in negative self-related thoughts and beliefs and significant increases in positive ones (38). These changes often predicted and/or mediated therapeutic outcomes. Similarly, changes in maladaptive self-beliefs during CBT have been found to predict subsequent changes in the severity of SAD symptoms (39). When it comes to personality, evidence suggests that psychological interventions may lead to changes in some traits. In particular, neuroticism and introversion may decrease following therapy (40–43). The sense of agency expressed in memory narratives, on the other hand, may increase (44, 45). This may result from interventions such as CBT contributing to increased fulfillment of psychological needs, including autonomy and mastery (46).

**Figure 1:**
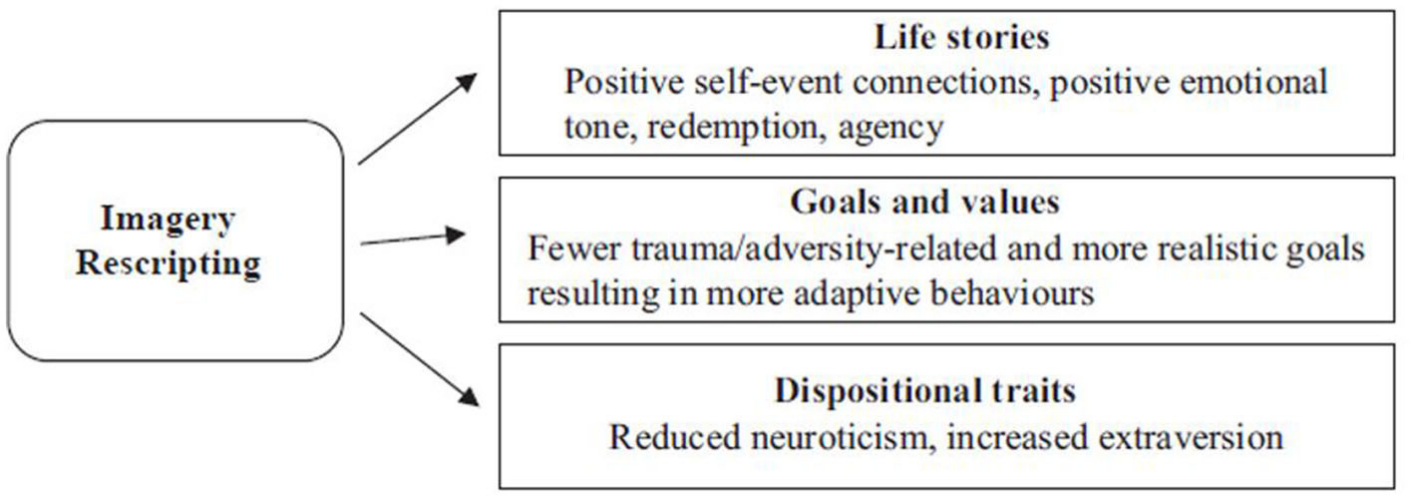
Narrative identity model of change in imagery rescripting. Adapted from Çili and Stopa (36). Copyright 2019 by S. Çili and L. Stopa. Reproduced by permission of Taylor and Francis Group, LLC, a division of Informa plc.

Based on the existing literature, our model (36) proposes that interventions like ImRs promote change at each personality layer (see Figure 1). First, ImRs promotes autobiographical reasoning. As a result of the memory reappraisal, patients make more benign self-event connections and possibly construct a redemptive story in which the aversive experience has a positive outcome. Because redemption involves positive affective shifts (35, 37), the rescripted memories may acquire a more positive emotional tone. At the same time, the new self-event connections, actively intervening in the memories, and imaginatively satisfying previously unmet needs may increase the sense of agency associated with these memories. Arntz and Weertman's (1) protocol explicitly incorporates need fulfillment and patients often employ memory intervention strategies which satisfy the needs they express in ImRs, including the need for autonomy [see (11)]. The increased sense of agency and mastery may contribute to recovery, as demonstrated by evidence that change in mastery of nightmare content mediated the efficacy of ImRs on nightmare frequency and distress (47). Second, since autobiographical reasoning may influence goal setting (48) and creating redemptive stories may lead to positive behavior change [see (49)], ImRs may also help patients set more realistic goals and abandon behaviors which helped maintain their symptoms (e.g., avoidance). This is why, as Brewin et al. (50) found, behavior change may occur spontaneously after ImRs. Third, changes in goals and in memories' meaning and affect may contribute to personality trait changes. Specifically, we propose that ImRs may contribute to reduced neuroticism and increased extraversion. This is because neuroticism is associated with negative affect (51), avoidance motivation (52), and more negative life chapters (53), whereas extraversion is associated with the opposite outcomes.

Personality changes may be amplified as individuals link the rescripted memories to other memories, modify further goals, and try to re-establish a sense of coherence in their life story and a sense of self-continuity and unity. They may, for example, reappraise other memories or new experiences in light of the rescripted memories' new meanings. Ultimately, these changes may influence the ways in which the rescripted memories – and potentially similar memories – exert their self and directive functions (36). In terms of the self-function, the new self-event connections could be incorporated into new or existing working selves which comprise more adaptive goals and self-images. The activation of these working selves following memory retrieval may then contribute to the rescripted memories' modified directive function as it elicits not only more positive self-images and self-evaluations, but also more positive affect and adaptive behaviors. In the long run, the frequent activation of the benign working selves may contribute to enhanced self-esteem and well-being [see (36)]. Taken together, these changes may account for the effectiveness of ImRs in reducing memory/imagery distress, targeting symptoms (3), and enhancing self-evaluations (8).

## Conclusion

Our model (36) takes a novel approach as it utilizes the narrative identity literature to elucidate the mechanisms of change in interventions like ImRs. Of course, this model has limitations. For example, Kazdin (54) argues that mechanisms of change need to be studied as mediators of treatment effects and the temporal precedence of mediator changes over outcome changes needs to be established. If ImRs is effective at reducing symptoms after 1 or few sessions (3) and personality traits require weeks or months to change (42), then trait changes may be simple treatment outcomes rather than mechanisms and may play a greater role in preventing relapse. Furthermore, we acknowledge that the empirical testing of our model is likely to be complex and that it may be difficult to establish whether even changes in the life story and goals are mechanisms or treatment effects. Following Kazdin's approach for assessing change would require multiple personality and symptom assessments, as well as long follow-ups, in order to understand the temporal sequence of the changes experienced by patients and whether the variables we have identified in our model mediate treatment effects. As the study of mechanisms in psychotherapy remains under scrutiny and there is a growing number of proposals on how to assess them [see (55–58)], determining the best way to test our model will also require further developments and consensus in this field.

Despite these limitations, we think that our model advances our understanding of ImRs. As the need to understand mechanisms of change in therapy becomes more pressing, we believe that it is essential for research into ImRs and similar interventions to recognize the complexity of personality. This may enable us to maximize the effectiveness of these interventions, delineate better when to use them, and identify who might relapse. Psychotherapy is an arena in which individuals make sense of their experiences and construct a coherent narrative identity (44, 59, 60). It may be time to recognize that through interventions like ImRs patients may rewrite their life story, not just isolated memories. The growing research on autobiographical memory, narrative identity, and mental health may provide some of the best tools for understanding this rewriting process.

## Author Contributions

All authors listed have made a substantial, direct, and intellectual contribution to the work and approved it for publication.

## Funding

The open access publication fee was provided by the University of Southampton.

## Conflict of Interest

The authors declare that the research was conducted in the absence of any commercial or financial relationships that could be construed as a potential conflict of interest.

## Publisher's Note

All claims expressed in this article are solely those of the authors and do not necessarily represent those of their affiliated organizations, or those of the publisher, the editors and the reviewers. Any product that may be evaluated in this article, or claim that may be made by its manufacturer, is not guaranteed or endorsed by the publisher.
